# Development of the major arboviral vector *Aedes aegypti* in urban drain-water and associated pyrethroid insecticide resistance is a potential global health challenge

**DOI:** 10.1186/s13071-019-3590-9

**Published:** 2019-07-08

**Authors:** Sinnathamby N. Surendran, Tibutius T. P. Jayadas, Kokila Sivabalakrishnan, Sharanga Santhirasegaram, Kalingarajah Karvannan, Thilini C. Weerarathne, S. H. P. Parakrama Karunaratne, Ranjan Ramasamy

**Affiliations:** 1 Department of Zoology, University of Jaffna, Jaffna, Sri Lanka; 2 Department of Zoology, University of Peradeniya, Peradeniya, Sri Lanka; 3 ID-FISH Technology Inc., Milpitas, CA 95035 USA

**Keywords:** *Aedes aegypti*, Arboviral diseases, Drain-water, Global health, Insecticide-detoxifying enzymes, Insecticide resistance, Mosquito vector biology

## Abstract

**Background:**

*Aedes aegypti* were found developing in the water in open public drains (drain-water, DW) in Jaffna city in northern Sri Lanka, a location where the arboviral diseases dengue and chikungunya are endemic.

**Methods:**

Susceptibilities to the common insecticides dichlorodiphenyltrichloroethane (DDT), malathion, propoxur, permethrin and deltamethrin and activities of the insecticide-detoxifying enzymes carboxylesterase (EST), glutathione S-transferase (GST) and monooxygenase (MO) were compared in adult *Ae. aegypti* developing in DW and fresh water (FW).

**Results:**

DW *Ae. aegypti* were resistant to the pyrethroids deltamethrin and permethrin, while FW *Ae. aegypti* were susceptible to deltamethrin but possibly resistant to permethrin. Both DW and FW *Ae. aegypti* were resistant to DDT, malathion and propoxur. Greater pyrethroid resistance in DW *Ae. aegypti* was consistent with higher GST and MO activities.

**Conclusions:**

The results demonstrate the potential for insecticide resistance developing in *Ae. aegypti* adapted to DW. Urbanization in arboviral disease-endemic countries is characterized by a proliferation of open water drains and therefore the findings identify a potential new challenge to global health.

## Background

*Aedes aegypti* mosquitoes are the primary vectors of many arboviral diseases that are of major global health concern [1–3], including chikungunya, dengue, Rift Valley fever, yellow fever and Zika. The adaptation of *Ae. aegypti* to blood-feed on humans and oviposit and undergo pre-imaginal development in water collections near human habitations has facilitated its global spread [4]. *Aedes aegypti* has long been regarded as developing only in fresh water (FW) collections near human habitations [5]. The closely related arboviral vector *Aedes albopictus* is a sylvatic species that has also adapted more recently to develop in freshwater habitats in the urban and semi-urban environment in many countries, including Sri Lanka [5–8]. Larval source reduction measures that target FW habitats therefore form the principal approach to controlling arboviral diseases transmitted by the two *Aedes* vectors worldwide [6]. However, *Ae. aegypti* and *Ae. albopictus* have recently been observed to also develop in brackish water collections in beach litter and coastal domestic wells [7–9], indicating a global need to extend larval control measures to such habitats.

**Fig. 1:**
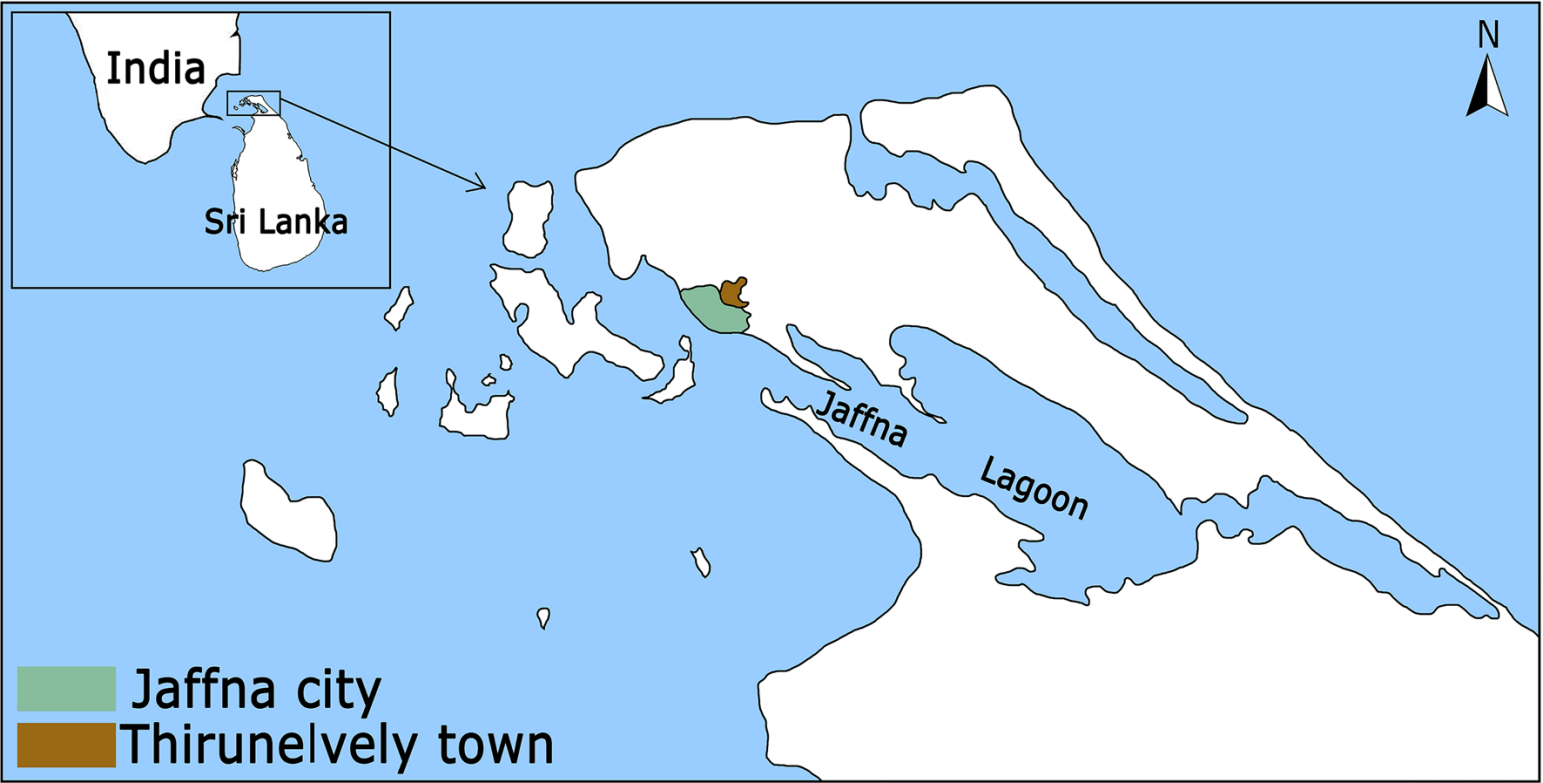
Map of Sri Lanka in relation to India showing Jaffna city, Jaffna lagoon and Thirunelvely town

**Fig. 2:**
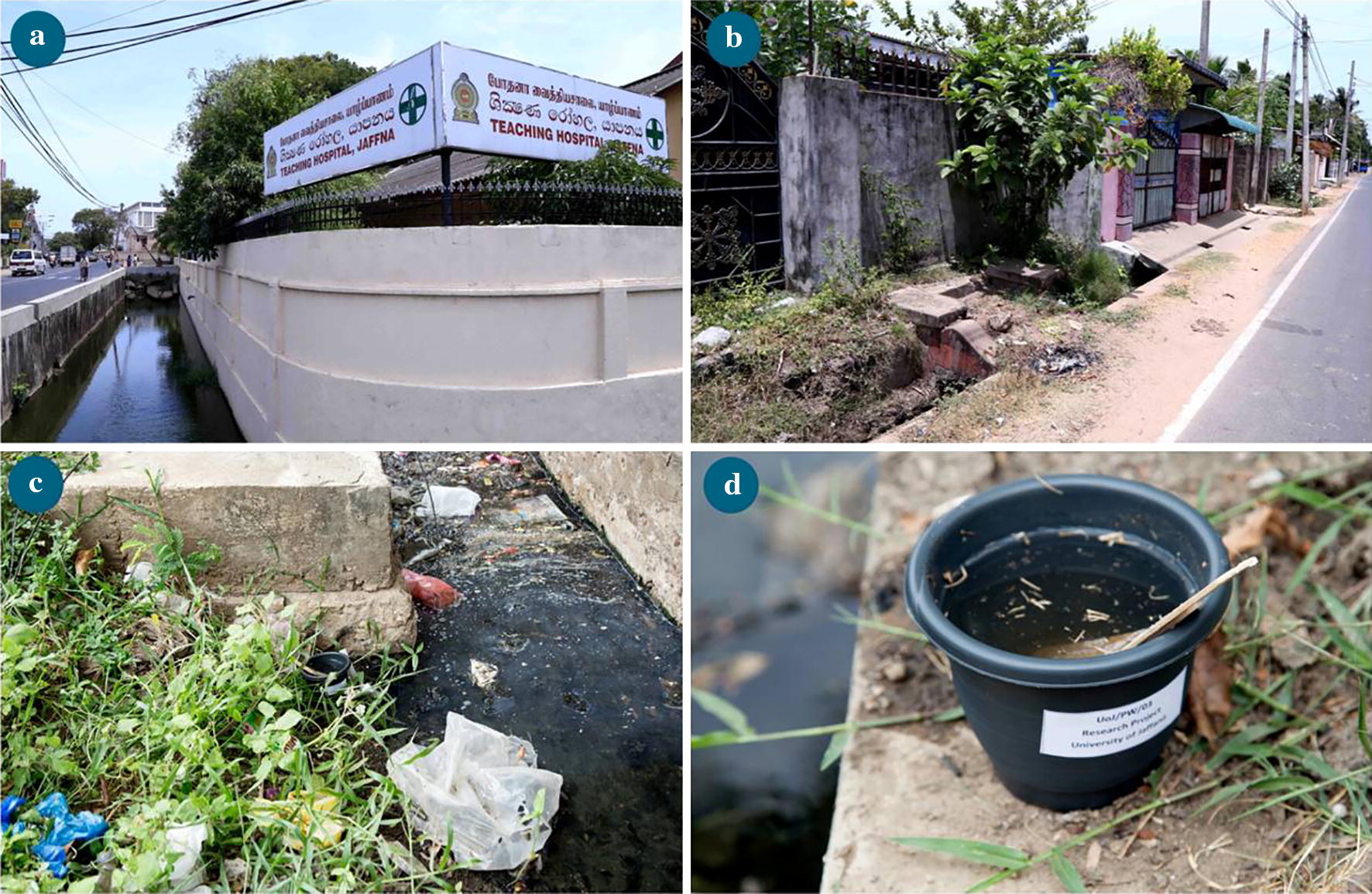
Drains with *Aedes aegypti* larvae in Jaffna city. **a** Open drain adjacent to the Jaffna Teaching Hospital (9°39′54.38″N, 80°1′0.17″E). **b**, **c** Open drains within residential areas of Jaffna city (9°40′49.76″N, 80°0′25.61″E; 9°40′23.43″N, 80°0′13.39″E). **d** Ovitrap for collecting DW larvae placed adjacent to an open drain and filled with water from the same drain (9°39′15.23″N, 80°1′15.22″E)

Open surface drains are common in urban areas of most arboviral disease-endemic, tropical and sub-tropical countries. There is a high incidence of dengue [7, 10] and chikungunya [11] in Jaffna city located in the densely populated Jaffna peninsula in northern Sri Lanka (Fig. 1). The Jaffna peninsula and Jaffna city do not have a sewer system and regulations require human waste to be treated in septic tanks within individual premises. Open surface drains are a feature of Jaffna (Fig. 2) and are the only means of draining rain-water and grey waste-water from homes and other premises into the Jaffna lagoon (Fig. 1). We investigated the presence of *Ae. aegypti* in open drains in Jaffna city, hypothesizing that the physiological mechanisms that might be developed to cope with pollutants in drain-water (DW) can collaterally generate greater resistance to insecticides with attendant implications for the control of arboviral diseases.

## Methods

### Study location and characteristics

The Jaffna peninsula is located at the northern tip of Sri Lanka in close proximity to India (Fig. 1). It receives an annual rainfall of 60–190 cm, mostly during the northeast monsoon between October and January with little or no rainfall during the rest of the year. The peninsula has a limestone geology and a population density of 700 persons per km^2^. Jaffna city, with an estimated population of 100,000, is the main urban centre in the peninsula. Water for domestic purposes is derived from individual household wells or piped from deep artesian wells located in Thirunelvely and contains a high concentration of calcium bicarbonate.

There is no sewerage system in Jaffna city or the rest of the Jaffna peninsula so that toilet waste-water is treated in septic tanks within individual premises and grey waste-water is discharged into open public drains that also collect excess rain water for final discharge into the Jaffna lagoon (Fig. 1). Waste-water from the Jaffna hospital is released into a public drain after aerobic treatment (Fig. 2a). Space spraying with either Pesguard^®^ (cyphenothrin and d-tetramethrin) or malathion is done once weekly inside the hospital premises for pest control. The public surface drains also receive waste grey water from households, business places and hotels. Plastic bags (Fig. 2c) and vegetation (Fig. 2b) can partially block the flow of water in drains, creating local stagnant pools that facilitate oviposition and pre-imaginal development of mosquitoes. Larvicides are not routinely applied to the drains. However, drains in the surroundings of houses where dengue infections have been identified are temporarily sprayed with temophos 1.1% w/w by public health officials. Larval source reduction is otherwise actively pursued by health officials all year round inside premises by the application of temephos to water tanks and encouragement of larvivorous fish culture in wells. Insecticides are, however, temporarily used for space and indoor residual spraying to control dengue outbreaks.

### Collection and establishment of laboratory colonies of *Ae. aegypti*

*Aedes aegypti* larvae were first observed in an open surface drain that receives grey waste-water from the Jaffna Teaching Hospital (9°39′11″N, 80°01′00″E) (Fig. 2a) and subsequently in other city drains. Fifteen ovitraps were positioned using water from the adjacent drain at 15 different collection sites in Jaffna city drains (Fig. 2d). Similarly, 15 ovitraps with piped fresh water were set up at 15 different sites in Thirunelvely town (9°41′05″N, 80°01′14′′E; Fig. 1). Ovitrap collections were done as previously described [7, 12]. The minimum distance between ovitraps was 150 m. Weekly larval collections were made from the ovitraps for 2 weeks. The DW and FW larvae from different ovitraps were separately pooled and maintained in the insectary in water collected fortnightly from the open drain outside the Jaffna hospital (Fig 1a) and Thirunelvely tap water, respectively, for establishing DW and FW colonies. Larvae were reared in 24 × 16 cm plastic trays with 1.5 l of water with a maximum number of 150 larvae per tray and fed with fish meal powder thrice a day. Emerging adults were identified and used to established separate self-mating colonies. About 150 adults (approximate female to male ratio of 3:2) of DW and FW origin were used to establish the colonies. The adults were maintained at 28–30 °C with a relative humidity of 75% and 12 h dark and light conditions. Adult females were blood-fed on mice. The DW and FW colonies were maintained up to 15 generations for experimental purposes in the laboratory in DW and FW, respectively, using previously described procedures [7].

### Determination of water quality parameters

Water quality parameters of the DW and FW used for colony maintenance were measured with a Multi Parameter Probe (Hanna Instruments, Leighton Buzzard, UK) according to the manufacturer’s instructions. The temperature, pH, dissolved oxygen (DO), electrical conductivity, total dissolved solids (TDS) and salinity were measured four times during larval generations 12 to 15.

### Bioassays for insecticide susceptibility

World Health Organization (WHO) procedures were followed to determine the insecticide susceptibility status of adult mosquitoes [13]. Non-blood-fed adult female mosquitoes of 3–4 days old from 12th to 14th generations of DW and FW colonies were tested with the WHO discriminating dosages of either 0.05% deltamethrin, 0.25% permethrin, 0.8% malathion, 0.1% propoxur or 4% DDT using WHO bioassay test kits. Two to three batches of 10–20 mosquitoes were exposed to insecticide impregnated papers for 60 min. At least 75 mosquitoes per replicate were tested per insecticide per rearing condition (DW or FW). Insecticide-exposed females were transferred into holding tubes and kept for 24 h and fed on 10% sugar solution. After a 24 h recovery period, mortality was determined and adjusted using Abbot’s formula [14] if the control mortalities were less than 20%. The WHO criteria were used to define a population susceptible (>98% mortality), suspected for resistance (90–98% mortality) and resistant (<90% mortality) [13].

### Enzyme assays

Unfed adult female mosquitoes of 3–4 days old from the 15th generation of the two colonies were subjected to biochemical assays using the WHO microplate method [15]. Eighty individual mosquitoes from each rearing condition were subjected individually to total protein, carboxylesterase (EST), glutathione S-transferase (GST) and monooxygenase (MO) assays [15].

### Statistical analysis

The two-tailed Student’s t-test at the 0.05 significance level for matched samples was performed to determine significant differences between DW and FW *Ae. aegypti* for insecticide survival and enzyme activities of GST, EST and MO.

## Results

### *Aedes aegypti* in Jaffna city drains and physical-chemical parameters of water used for rearing drain-water and fresh-water laboratory colonies

Of the 121 sites along open public drains with areas of stagnant water inspected in Jaffna city, eight were observed to contain *Ae. aegypti* larvae. Self-mating laboratory colonies of DW and FW *Ae. aegypti* were established with larvae collected from the DW and FW ovitraps that were established as described and then maintained for 15 generations in the respective water sources. The observed water quality parameters of temperature, pH, dissolved oxygen, conductivity, total dissolved solids and salinity of DW and FW used for rearing colonies are shown in Table 1. FW contained significantly higher DO and significantly lower TDS than DW, while DW had significantly higher salt and electrical conductivity than FW (Table 1).

**Table 1:** Water quality parameters of the experimental colonies. Results are the mean and standard deviation (SD) of four measurements made during larval generations 12 to 15

Parameter	Fresh water Mean ± SD	Drain water Mean ± SD	Comparison
Temperature (°C)	29.5 ± 0.4	29.3 ± 0.4	*t* = 0.49; *P* = 0.322
pH	7.4 ± 0.2	7.2 ± 0.5	*t* = 0.48; *P* = 0.326
Dissolved oxygen (ppm)	3.09 ± 0.20	1.53 ± 0.15	*t* = 10.85; *P* = 0.001
Conductivity (μS/cm)	1518 ± 69	4890 ± 387	*t* = 14.85; *P* = 0.001
Total dissolved solids (ppm)	774.33 ± 66.40	2577.67 ± 185.05	*t* = 15.88; *P* = 0.001
Salinity (PSU)	0.77 ± 0.07	2.68 ± 0.34	*t* = 9.44; *P* = 0.001

*Abbreviations*: ppm, parts per million; μS/cm, micro-Siemens per centimeter; PSU, practical salinity unit equivalent to parts per thousand of salt

### Adult insecticide susceptibility bioassays

**Fig. 3:**
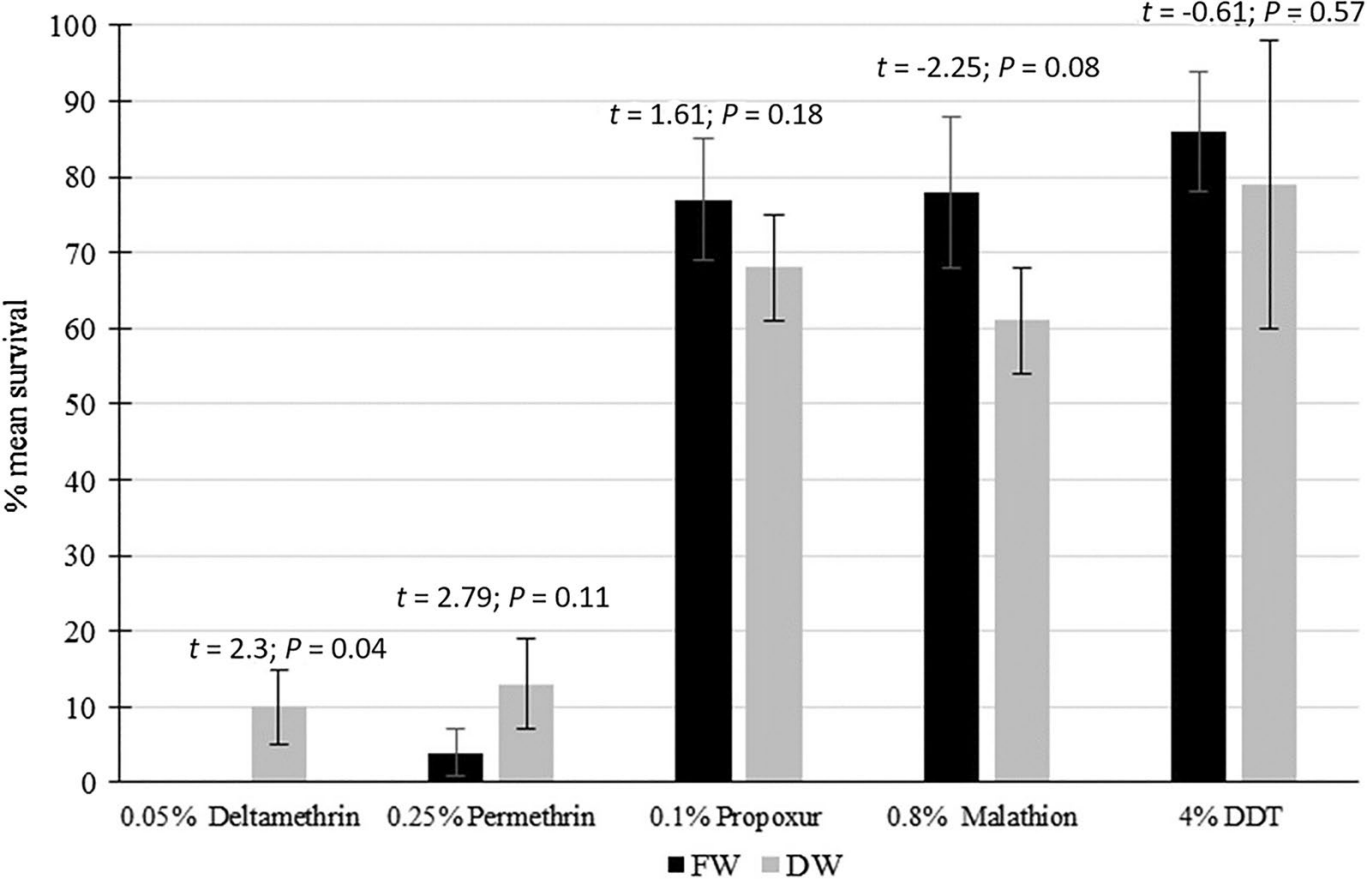
Susceptibility of DW and FW *Aedes aegypti* to different insecticides. The results show the mean percentage survival ± standard deviation. S, susceptible (≥98% mortality); R, confirmed resistance (<90% mortality); V, possible resistance and verification needed (90–97% mortality); according to WHO test guidelines [13]. The error bars represent standard deviations. The calculated t-test statistic and probability *P*-value for matched samples are shown above the bars

Figure 3 shows that both DW and FW *Ae. aegypti* were resistant (<90% mortality) to the WHO recommended test concentrations of DDT (an organochlorine insecticide), malathion (an organophosphate) and propoxur (a carbamate). There were no statistically significant differences in resistance to propoxur, DDT and malathion between the FW and DW mosquitoes. Conversely, FW *Ae. aegypti* were totally susceptible (0% survival) to deltamethrin (a Type II pyrethroid) to which DW *Ae. aegypti* were resistant (13% survival), and this difference was statistically significant. FW *Ae. aegypti* were classified as possibly resistant (4% survival) to permethrin (a Type I pyrethroid) while the DW *Ae. aegypti* were resistant (10% survival), but this tendency for a difference was not statistically significant.

### Activities of potential insecticide detoxifying enzymes

Activities of the detoxifying enzymes carboxylesterase, glutathione S-transferase and monooxygenase in DW and FW *Ae. aegypti* are presented in Table 2. DW *Ae. aegypti* had more than threefold higher activities of GST and cytochrome P450-dependent MO than FW *Ae. aegypti* that were significantly different, while EST activities in DW and FW *Ae. aegypti* were not significantly different.

**Table 2:** Activities of potential insecticide detoxifying enzymes in fresh and drain water *Aedes aegypti.* Enzyme activities are expressed as the mean carboxylesterase (EST) activity (µmo min^−1^ mg^−1^), mean glutathione S-transferase (GST) activity (µmo min^−1^ mg^−1^) and mean monoxygenase (MO) quantity (equivalent units of cytochrome P450 per mg). Eighty mosquitoes were individually assayed for each determination

Colony	Mean enzyme activity ± SE		
	GST	EST	MO
FW	0.09 ± 0.02	0.08 ± 0.01	0.006 ± 0.001
DW	0.33 ± 0.02	0.09 ± 0.01	0.020 ± 0.002
Comparison	*t* = 8.12; *P* < 0.001	*t* = 0.77; *P* = 0.222	*t* = 3.89; *P* < 0.001

*Abbreviation*: SE, standard error of the mean

## Discussion

There are no specific anti-viral drugs to treat the major arboviral diseases and a universally approved vaccine is presently only available for yellow fever. Therefore, controlling vector populations is currently the main approach for combating arboviral diseases. However, resistance in *Ae. aegypti* to the four widely used classes of insecticides that act on the insect nervous system (carbamates, organochlorines, organophosphates and pyrethroids) has developed in many parts of the world [16]. Resistance to insecticides can involve reduced cuticle penetration, sequestration from target sites, mutations in target receptors and the active site of target enzymes, and increased activity or overproduction of enzymes that detoxify insecticides [17]. Many of these resistance mechanisms have now been documented in *Ae. aegypti* [16, 18]. The present study only measured activities of potential insecticide detoxifying enzymes but the other possible resistance mechanisms in DW-adapted *Ae. aegypti* also merit investigation.

The study involved pooling *Ae. aegypti* collected from several different drain habitats for subsequent maintenance in DW from one selected habitat for up to 15 generations in the laboratory, a procedure that can result in adaptation of *Ae. aegypti* to DW under more uniform conditions than is possible under field conditions. The mixing of FW and DW *Ae. aegypti* can occur in the different field sites in Jaffna city but not in the laboratory. The relative proportions of FW and DW *Ae. aegypti* in Jaffna city is likely to depend of the relative availability of FW and DW habitats and how larval control measures are applied in the city. Extreme reduction of FW larval sources can lead to a predominance of DW *Ae. aegypti* in the city. A limitation of our study was that adults emerging from larvae collected in the field from FW and DW habitats were not tested in insecticide resistance studies. Obtaining an adequate number of DW adults for such tests would have necessitated establishing laboratory colonies for at least a few generations that would have resulted in some attendant adaptation to DW in any case. Possible studies with recently adapted *Ae. aegypti* is also complicated by the many different DW habitats in the city that varied in the physical and chemical characteristics of their water.

The history of insecticide use is pertinent to understanding the present status of insecticide resistance in *Ae. aegypti* in the Jaffna peninsula. Controlling anopheline populations to reduce the prevalence of malaria was the most important objective of mosquito vector control programs in Sri Lanka until the elimination of malaria in 2013 [19]. DDT was first introduced after the Second World War for indoor residual spraying (IRS) throughout the island and was initially very effective in controlling malaria. It was replaced by Malathion in 1977 due to the development of widespread DDT resistance in the anopheline malaria vectors. Malathion continued to be used for IRS in the Jaffna peninsula until 2009 when it was replaced with deltamethrin [19]. Island-wide IRS was discontinued in 2013 after the elimination of malaria. Insecticide use for dengue control in Jaffna city entails the regular application of temephos (an organophosphate) to freshwater collections in water tanks and the use of larvivorous fish in wells within individual premises to control pre-imaginal stages of *Ae. aegypti*. The localized space spraying (fogging) and IRS with pyrethroids are also carried out targeting adult vectors for controlling outbreaks of dengue. Public open drains are not routinely treated with insecticides except that temephos is temporarily applied to drains surrounding premises where dengue patients have been identified. Organophosphate and carbamate insecticides are used as agricultural pesticides while pyrethroids are used in mosquito coils and pyrethroid-impregnated bednets in the Jaffna peninsula and elsewhere in Sri Lanka.

EST, GST and cytochrome P450-dependent MO are enzymes that normally perform essential physiological functions in mosquitoes. However, increased activities of GST and MO can inactivate organochlorine insecticides, EST and GST the organophosphates, and GST and MO the pyrethroids [17, 20, 21]. The higher GST and MO activities in DW *Ae*. *aegypti* compared with FW *Ae*. *aegypti* is consistent with their observed greater resistance to the two common pyrethroid insecticides deltamethrin and permethrin.

High levels of resistance to DDT, many years after the use of DDT for IRS was ceased, has been reported for malaria vectors in Sri Lanka [21, 22]. The most recent previous study on insecticide resistance in FW *Ae. aegypti* collected in Thirunelvely documented resistance to DDT and malathion, possible resistance to permethrin and susceptibility to propoxur in 2013 [23]. As proposed for malaria vectors in the country [24], DDT and malathion resistance in Jaffna *Ae. aegypti* is probably the result of the intense use of DDT and malathion for malaria control prior to 2009. The resistance to propoxur that has occurred since 2013 may have been caused by the resumption of intensive agriculture with use of carbamate and organophosphate pesticides in the Jaffna peninsula after 2009. FW *Ae. aegypti* in Jaffna were reported to have some of the lowest activities of EST, GST and MO in the country in 2013 [23]. The present results show that DW *Ae. aegypti* have significantly higher GST and MO activity than FW *Ae. aegypti* which could have arisen through an adaptation to detoxify organic pollutants present in DW that are absent in FW. DW used for collecting and maintaining DW *Ae. aegypti* is likely to be contaminated with chemicals in the form of soaps as well as aromatic hydrocarbon detergents and disinfectants. Contamination of DW with animal and even human excreta is also possible, although the city regulation that human excreta are treated in septic tanks within premises is rigidly enforced. GST and MO can detoxify aromatic compounds, including pyrethroids, by helping to transform them to more soluble compounds that can be more readily excreted [17].

It is not possible to exclude the possibility that traces of pyrethroid insecticides used within the hospital or nearby homes and leaching into DW also contribute to the observed pyrethroid resistance in DW *Ae. aegypti*. If this is the case, inheritable mutations selecting for insensitivity in the axonal voltage-gated sodium channel (VGSC) target of pyrethroids may contribute to the greater pyrethroid resistance in DW *Ae. aegypti* in addition to elevated GST and MO activities [16, 18, 20]. *Aedes aegypti* possesses multiple copies of *gst* (coding for GST) and *cyp* genes (coding for MO) and regulation of their overexpression in pyrethroid resistance is complex and incompletely understood at present [25, 26].

*Aedes aegypti* is now known to be capable of adapting to oviposit and undergo pre-imaginal development in different types of anthropogenic habitats [7–10, 27–30] but such adaptation has not previously been related to insecticide resistance in adult mosquitoes. Both transient and inheritable changes in larval salinity tolerance were observed in *Ae. aegypti* adapting to salinity [31, 32]. Increased activities of GST and MO were reported in *Ae. aegypti* larvae transiently exposed to sub-lethal concentrations of the polycyclic aromatic hydrocarbon fluoranthene, which is found in road tar residues, but the studies were not extended to adult mosquitoes or subsequent generations to investigate heritability [33, 34]. Relationships between environmental chemicals and insecticide resistance have been reported for malaria vectors [35]. Resistance in the *Anopheles gambiae* complex to permethrin, differential cuticular protein synthesis, as well as higher GST and MO activities have been associated with oil spills in Nigeria [36, 37], and resistance to deltamethrin with the use of agricultural chemicals in Tanzania [38].

## Conclusions

The adaptation of mosquito vectors of human disease to a variety of novel habitats created by anthropogenic activities can lead to range expansion [27]. The results of the present study are consistent with the hypothesis that physiological mechanisms developed to cope with chemicals present in urban DW during pre-imaginal development can generate greater resistance to common pyrethroids in adult mosquito vectors. Greater resistance to pyrethroid insecticides in adult DW *Ae. aegypti* has particularly important ramifications because pyrethroids are an important class of insecticides used to control adult *Ae. aegypti* [39, 40]. Since open drains for grey waste-water are ubiquitous features of densely populated urban areas in parts of the world where arboviral diseases are endemic, the present findings raise the possibility that the drains can become sources for selecting mosquito vectors that are resistant to pyrethroid insecticides leading to a subsequent expansion of their range to surrounding areas [27]. This poses a challenge to controlling mosquito-borne diseases, not only in Jaffna, but in many other disease-endemic tropical and subtropical countries. Surface drains are also associated with other safety hazards. Measures therefore need to be taken to build appropriate drains underground in urban areas of disease-endemic countries.

## Data Availability

All data generated during this study are included in this published article.

## Abbreviations

DDT: dichlorodiphenyltrichloroethane
DO: dissolved oxygen
DW: drain-water
EST: carboxylesterase
GST: glutathione S-transferase
FW: freshwater
IRS: indoor residual spraying
MO: monooxygenase
TDS: total dissolved solids
VGSC: voltage-gated sodium channel
WHO: World Health Organization
